# A Rare Case of Pregnancy-Associated Thrombotic Thrombocytopenic Purpura: Challenges in Diagnosis and Management

**DOI:** 10.7759/cureus.83587

**Published:** 2025-05-06

**Authors:** Fatemah Albousaeed, Anwar AlmohammedAli, Zainab Alowainati, Zainab Salman, Mohammed Alsubhi

**Affiliations:** 1 College of Medicine and Health Sciences, Arabian Gulf University, Manama, BHR; 2 College of Medicine, Jordan University of Science and Technology, Irbid, JOR; 3 College of Medicine, Cairo University, Cairo, EGY; 4 Family Medicine, King Abdulaziz Medical City, Jeddah, SAU

**Keywords:** adamts13 deficiency, differential diagnosis, hellp syndrome, maternal morbidity, microangiopathic hemolytic anemia, plasma exchange, preeclampsia, pregnancy-associated ttp, thrombocytopenia, thrombotic thrombocytopenic purpura

## Abstract

Thrombotic thrombocytopenic purpura (TTP) in pregnancy is a rare but potentially life-threatening condition that presents significant diagnostic challenges due to its overlapping features with other pregnancy-related disorders, such as preeclampsia and HELLP (hemolysis, elevated liver enzymes, and low platelet count) syndrome. We present the case of a 29-year-old gravida 2 para 1 woman at 35 weeks of gestation who presented with generalized weakness, nausea, and bruising, with laboratory findings indicating thrombocytopenia, microangiopathic hemolytic anemia, and schistocytes on peripheral blood smear. A diagnosis of pregnancy-associated TTP was made after excluding other causes such as preeclampsia. The patient was treated with plasma exchange, and her condition improved, leading to a successful cesarean section at 36 weeks of gestation. The neonatal outcome was favorable. This case highlights the importance of early recognition of TTP in pregnancy, as timely diagnosis and treatment with plasma exchange are critical for both maternal and fetal survival.

## Introduction

Thrombotic thrombocytopenic purpura (TTP) is a rare but potentially life-threatening hematologic disorder characterized by the pentad of thrombocytopenia, microangiopathic hemolytic anemia, neurological symptoms, renal dysfunction, and fever. The pathophysiology of TTP is primarily attributed to a deficiency in the ADAMTS13 enzyme, which normally cleaves von Willebrand factor, preventing the formation of microthrombi [1,2]. Although TTP is a medical emergency, its diagnosis can be challenging, especially in pregnancy, where its clinical presentation can overlap with other conditions such as preeclampsia and HELLP (hemolysis, elevated liver enzymes, and low platelet count) syndrome [1-3].

Pregnancy-associated TTP is exceedingly rare, with an incidence of approximately 1 in 25,000 pregnancies [4]. It typically presents in the third trimester, though it can occur at any stage of pregnancy, and may be triggered by factors such as infection, autoimmune disorders, or, in rare cases, pregnancy itself. Early diagnosis and treatment are critical to preventing maternal and fetal complications. Plasma exchange remains the cornerstone of treatment, and timely intervention has been associated with improved maternal outcomes [5,6]. Despite its rarity, awareness of pregnancy-related TTP is essential for obstetricians and hematologists to ensure appropriate management and improve the prognosis for both mother and infant.

## Case presentation

A 29-year-old gravida 2 para 1 woman, at 35 weeks of gestation, was admitted to the obstetrics ward with a history of generalized weakness, nausea, and bruising over the past week. She had noted easy bruising on her arms and legs, which progressed to petechial rash and visible purpura. Additionally, she reported a mild headache and dizziness. Her obstetric history was significant for a previous uncomplicated vaginal delivery, and she was otherwise healthy with no known medical comorbidities. There was no history of recent trauma, medications (other than routine prenatal vitamins), or infections.

The patient’s obstetric course was unremarkable until the current pregnancy. Prenatal screening had been normal, and her prenatal visits were regularly attended with no complications. She denied any recent fever, chills, or changes in fetal movement. Family history was non-contributory for bleeding disorders or thrombotic events.

On examination, the patient appeared acutely ill but was afebrile. Blood pressure was 120/70 mmHg, and heart rate was 98 beats per minute. She had a palpable purpuric rash extending from her upper limbs to her lower limbs, with numerous petechiae scattered across her chest and abdomen. No mucosal bleeding or signs of active hemorrhage were noted. Neurological examination revealed mild dizziness but no focal deficits. Abdominal examination revealed a gravid uterus, and fetal heart sounds were audible with normal rhythm and rate. There were no signs of preeclampsia, such as proteinuria or hyperreflexia. Her cardiovascular and respiratory examinations were otherwise normal.

Initial laboratory investigations revealed a hemoglobin level of 10.2 g/dL (normal: 11-14 g/dL), a platelet count of 32,000/µL (normal: 150,000-400,000/µL), and a normal white blood cell count. Coagulation studies showed a prothrombin time (PT) of 12.4 seconds (normal: 11-13 seconds) and an activated partial thromboplastin time (aPTT) of 33 seconds (normal: 30-40 seconds). Peripheral blood smear showed schistocytes, confirming microangiopathic hemolytic anemia. Reticulocyte count was elevated at 8.5%, indicative of bone marrow response to anemia. Lactate dehydrogenase (LDH) was markedly elevated at 1,250 U/L (normal: 140-280 U/L), and haptoglobin was low at 12 mg/dL (normal: 30-200 mg/dL), further suggesting hemolysis. Renal function tests revealed a serum creatinine of 0.8 mg/dL, and liver enzymes were normal.

Given the combination of thrombocytopenia, microangiopathic hemolytic anemia, and the presence of schistocytes, a diagnosis of TTP was suspected. However, in the context of pregnancy, the diagnosis posed a challenge due to the overlapping clinical features with other obstetric conditions, including preeclampsia and HELLP syndrome. To rule out preeclampsia, the patient's blood pressure remained stable, and there was no proteinuria or hepatic involvement, making it less likely. Furthermore, the normal liver function and absence of elevated liver enzymes ruled out HELLP syndrome. The presence of microangiopathic hemolytic anemia with thrombocytopenia and elevated LDH led to the final diagnosis of pregnancy-associated TTP.

A decision was made to initiate treatment with plasma exchange, the gold standard for TTP management. This was carried out promptly, with five sessions of plasmapheresis over the following week. A multidisciplinary team, including obstetricians, hematologists, and neonatologists, was consulted to manage the patient's condition in coordination with fetal well-being. Due to the late stage of pregnancy, the decision was made to deliver the fetus via cesarean section following improvement in the patient's platelet count and stabilization of her condition. Delivery occurred at 36 weeks of gestation without any complications. The newborn was admitted to the neonatal intensive care unit for observation but showed no signs of acute distress.

Postpartum, the patient's platelet count normalized, and she had a gradual improvement in symptoms, including resolution of bruising and purpura. The patient was discharged on day 7 postpartum with normal platelet counts and stable hemoglobin levels. She was closely monitored for any recurrence of TTP symptoms and was advised to follow up with hematology for further evaluation. At her six-week postpartum follow-up, the patient had no recurrence of TTP symptoms, and her blood counts remained within normal limits.

## Discussion

TTP in pregnancy is an exceptionally rare but critically important condition that poses significant challenges in diagnosis and management. This case report highlights the diagnostic complexities of pregnancy-associated TTP, emphasizing the need for heightened clinical awareness and a systematic approach to diagnosis [1-4]. Although TTP is infrequently seen in pregnancy, it is crucial to recognize it early due to its potential for severe maternal and fetal morbidity and mortality. This case underscores the importance of distinguishing TTP from other pregnancy-related disorders with similar clinical features, such as preeclampsia and HELLP syndrome, both of which are more commonly encountered during pregnancy.

The classic pentad of TTP includes thrombocytopenia, microangiopathic hemolytic anemia, neurological symptoms, renal dysfunction, and fever [6]. However, it is important to note that not all patients with TTP will present with all five features, and the absence of fever or neurological symptoms should not exclude the diagnosis. In this case, the patient's presentation of thrombocytopenia, microangiopathic hemolytic anemia, and schistocytes on peripheral blood smear was key to suspecting TTP. However, the overlapping symptoms with preeclampsia, such as headache, dizziness, and thrombocytopenia, complicated the diagnostic process. The absence of elevated liver enzymes and the presence of schistocytes, along with a markedly elevated LDH level, helped differentiate TTP from HELLP syndrome, which often presents with elevated liver enzymes [5-8].

The pathophysiology of TTP in pregnancy is not fully understood but is thought to involve a deficiency of the ADAMTS13 enzyme, which is responsible for cleaving von Willebrand factor and preventing platelet aggregation. In the setting of pregnancy, factors such as increased levels of von Willebrand factor, immunological changes, or the activation of the complement system may contribute to the development of TTP [1-4]. Pregnancy-associated TTP can be triggered by various factors, including infection, autoimmune diseases, or, rarely, the pregnancy itself. This case is particularly noteworthy because it demonstrates that pregnancy, especially in the third trimester, can be a trigger for TTP in the absence of other predisposing factors.

One of the most critical aspects of managing TTP in pregnancy is the prompt initiation of plasma exchange, which remains the cornerstone of treatment. Plasma exchange works by removing the pathological autoantibodies against ADAMTS13 and replacing the deficient enzyme with normal plasma, significantly improving outcomes in both maternal and fetal survival. In this case, the patient responded well to plasma exchange, with normalization of platelet counts and resolution of the purpura and anemia [3,8]. The decision to deliver the fetus via cesarean section at 36 weeks of gestation was made after the patient’s condition stabilized. Although there is some evidence to suggest that early delivery may improve maternal outcomes in severe cases of TTP, it should be balanced with the risks of preterm birth. In this case, the neonatal outcome was favorable, and the infant did not require any major interventions.

The challenges in diagnosing TTP during pregnancy arise from the similarity of its clinical features to other obstetric complications. Preeclampsia, which presents with hypertension, proteinuria, and thrombocytopenia, is the most common differential diagnosis. However, in this patient, the absence of hypertension, proteinuria, and significant liver involvement helped rule out preeclampsia and HELLP syndrome. The presence of microangiopathic hemolytic anemia with schistocytes, low haptoglobin, and elevated LDH pointed towards TTP as the more likely diagnosis. As such, this case reinforces the need for careful differential diagnosis, as well as the importance of considering TTP even in the absence of the full classical pentad.

In addition to plasma exchange, supportive care is vital in the management of TTP. Antiplatelet therapy, such as aspirin and corticosteroids, may be considered in some cases, but their role in pregnancy-associated TTP remains unclear and should be approached with caution [5-9]. In this patient, the use of plasma exchange was successful, and no additional pharmacologic agents were required. Close monitoring of renal function, platelet count, and hemoglobin levels is essential throughout the treatment course.

Given the rarity of pregnancy-associated TTP, there are limited large-scale studies to guide clinical management. Much of the current knowledge comes from case reports and small series, underscoring the importance of sharing clinical experiences to advance understanding and improve outcomes [7,8]. Early recognition, a high index of suspicion, and prompt initiation of plasma exchange are critical in preventing life-threatening complications. This case report contributes to the growing body of literature on TTP in pregnancy and reinforces the need for ongoing vigilance in diagnosing and managing this rare disorder.

Long-term follow-up is necessary to monitor for any recurrence of TTP or the development of other pregnancy-related complications [9]. In this case, the patient had an uneventful postpartum course, with no recurrence of TTP symptoms at her six-week follow-up. However, some studies suggest that patients who experience TTP in pregnancy may be at increased risk of recurrence in future pregnancies, particularly in the setting of inherited forms of TTP. Therefore, genetic counseling and careful monitoring during subsequent pregnancies are advised.

## Conclusions

In conclusion, TTP in pregnancy, although rare, presents significant diagnostic and management challenges due to its overlapping features with other common pregnancy-related disorders like preeclampsia and HELLP syndrome. Early recognition of TTP, supported by appropriate laboratory findings such as microangiopathic hemolytic anemia, thrombocytopenia, and schistocytes, is crucial for initiating prompt treatment with plasma exchange, which remains the cornerstone of management. Timely intervention improves maternal outcomes and can help ensure fetal survival, as demonstrated in this case. Given the rarity of pregnancy-associated TTP, further research is necessary to refine diagnostic criteria and management strategies, and to improve understanding of its pathophysiology and long-term implications for both mother and child. This case underscores the importance of a high index of suspicion, early diagnosis, and multidisciplinary care in managing this life-threatening condition.
